# Application of community dialogue approach to prevent adolescent pregnancy, early marriage and school dropout in Zambia: a case study

**DOI:** 10.1186/s12978-022-01335-8

**Published:** 2022-01-31

**Authors:** Ireen Zamanga Zulu, Joseph Mumba Zulu, Joar Svanemyr, Charles Michelo, Wilbroad Mutale, Ingvild Fossgard Sandøy

**Affiliations:** 1 Departments of Health Policy and Health Promotion and Education, School of Public Health, University of Zambia, PO Box 50110, Lusaka, Zambia; 2 Centre for International Health, University of Bergen, Årstadveien 21, Postboks 7804, 5020 Bergen, Norway; 3 Department of Epidemiology and Biostatistics, School of Public Health, University of Zambia, PO Box 50110, Lusaka, Zambia; 4 Department of Health Policy and Management, School of Public Health, University of Zambia, PO Box 50110, Lusaka, Zambia

**Keywords:** Adolescent pregnancy, Early marriage, Community dialogue, Sexual and reproductive health, School dropout, Poverty

## Abstract

**Background:**

Adolescent pregnancy carries both health and economic risks for the pregnant girl and resulting baby, and it is common in Zambia. Providing alternative methods of preventing early pregnancy than abstinence is regarded as culturally and religiously unacceptable in most parts of the country. The community dialogue approach is being tested to address norms and beliefs around early pregnancy, marriage and school dropout, and is based on Paulo Freire’s transformative communication approach. The objective of this paper was to understand parents’ perspectives on the application of the community dialogue approach in addressing adolescents’ early pregnancy and school dropout in a cluster randomized controlled trial in rural Zambia.

**Methods/design:**

This was a case study design. We nested the study in the community dialogue intervention arm of the Research Initiative to Support the Empowerment of Girls trial in Zambia. Dialogue meetings were held and economic support was provided for a period of 27 months from September 2016 until November 2018. We held focus group discussions in November 2018 with guardians/parents in six schools in Chibombo District of Central Province. All the discussions were audio recorded and transcribed verbatim. Thematic analysis was used to analyze the data.

**Results:**

The guardians/parents perceived the community dialogue to be a relevant approach for addressing social and cultural norms regarding early pregnancy, marriage and school dropout. It was embraced for its value in initiating individual and collective change. The facilitators’ interactive approach and dialogue in the community meetings coupled with the use of films and role plays with the parents, lead to active participation and open discussions about sexual and reproductive health (SRH) topics during the community dialogue meetings. Group interactions and sharing of experiences helped parents clarify their SRH values and subsequently made them feel able to communicate about SRH issues with their children. However, cultural and religious beliefs among the parents regarding some topics, like the use of condoms and contraceptives, complicated the delivery of reproductive health messages from the parents to their children.

**Conclusion:**

The study indicated that the community dialogue was appreciated by the parents and helped in addressing cultural barriers to discussing SRH issues between generations.

## Plain language summary

Adolescent pregnancy carries health and economic risks for the pregnant girl and her baby. However, many adolescent girls in Zambia become pregnant and this is partly due to social norms around fertility and marriage. Community dialogue meetings may be useful in order to discuss norms and beliefs around early pregnancy, marriage and school dropout and encourage reflection around cultural practices in the communities. A cluster randomized controlled trial in rural Zambia tested the effects of organizing community dialogue meetings and providing economic support for families with adolescent girls for a period of 27 months, from 2016 to 2018. The objective of this case study was to understand parents’ perspectives on the use of the community dialogue approach.

Focus group discussions were held in November 2018 with guardians/parents in six schools in Chibombo District of Central Province. The guardians/parents perceived the community dialogue to be a relevant approach for addressing social and cultural norms regarding early pregnancy, marriage and school dropout. The facilitators’ interactive approach in the community meetings coupled with the use of films and role plays, lead to active participation and open discussions about sexual and reproductive health topics during the community dialogue meetings. Group interactions and sharing of experiences made the guardians feel able to communicate about sexual and reproductive health issues with their children. However, cultural and religious beliefs among the parents regarding the use of condoms and contraceptives made it difficult for them to touch upon this topic.

## Background

According to WHO about 7 million adolescent girls give birth before the age of 18 years every year. Most of these births happen in low and middle income countries [1]. In addition, annually 150,000 young people aged 10–19 years are infected with HIV according to 2020 data [2]. Young people’s limited exposure to sexual reproductive health (SRH) information is among the factors leading to this situation [3–5]. Data from sub-Saharan Africa suggest that while a significant number of adolescents have their first sexual experience before age 15 (ranging from 2.0 to 27.0%) [6], many of them do not use any form of protection to prevent pregnancy or sexually transmitted infections [7, 8].

The need to strengthen efforts to prevent pregnancy among adolescent girls in sub-Saharan Africa has been increasingly recognized over recent years [9–11]. In Zambia the high prevalence of child marriage and teenage pregnancy greatly contribute to high fertility and population growth, and is closely interrelated with a range of economic and social determinants [12–14]. Data from Ministry of Education, Science, Vocational Training and Early Education shows that on average there are over 15,000 pregnancies reported each year among schoolgirls, and more than 80% of these pregnancies occur in rural areas [15]. The girls in the poorest quintile of Zambian household are five times more likely to be married before the age of 18 years than those in the wealthiest quintile households [16]. Data from the 2018 Demographic and Health Survey indicate that there has been little change in the national prevalence of child marriages since 2002 [16].

Numerous prevention strategies such as health education, skills‐building and improving accessibility to contraceptives have been employed by countries across the world, in an effort to address early pregnancy [17–20]. However, behaviors are influenced by societal norms, and many of these prevention strategies do not address the social norms that endorse or contribute to early pregnancy, such as girls being regarded as ready for marriage when they drop out of school as long as they have reached menarche, even if they are very young [20–23].

The community dialogue or community conversation (CC) approach appears to be a promising approach for addressing social and cultural norms regarding early pregnancy, marriage and school dropout [24, 25]. The facilitator should not attempt to teach or advise community members. Rather his or her role is to bring out pre-existing community understanding by asking open questions to trigger reflection and strengthen or encourage the community to analyse and solve local problems [26, 27]. As problems and solutions are discussed, the facilitator guides the discussion and participates in developing concerted action plans [24]. The approach is based on Paulo Freire’s theory and may involve cultural themes in the form of materials such as pictures, comics, short stories, songs, videos and dramas that are used to generate discussion [28–30]. This approach works better if it takes up practical and immediate pressing issues in a community before moving on to deeper, systemic or taboo issues [31, 32]. Studies have indicated that the CC method can play a significant role in contributing to intended change processes in HIV and AIDS-related community attitudes and practices [30, 33, 34]. Community dialogue and community conversation terms will be used synonymously in this article.

Evidence indicates that ensuring adolescents have correct information about sexual and reproductive health can be vital to preventing early child marriages and school dropout [3, 35, 36]. Teenagers obtain information on sexual behaviour from various sources [3, 35, 36] but parents are in most cultures regarded as the primary educators for children when it comes to sexuality education [32, 37, 38]. However, the nature and quality of the relationship between teenagers and their parents or guardians can have a major influence on the decisions that the teenagers make about sex [37, 38]. Parents who set and enforce rules, monitor behaviour and provide support can have a positive impact on teenage sexual behaviour [32, 37, 38]. Communication between parents and adolescent girls protects the latter from engaging in risky sexual behaviour and associated adverse health consequences [39]. Parents should talk openly about sex with their children, and in order to do this, they may need to be empowered with both knowledge and skills in sex education. Thus some sexuality education programmes foster strong parent–child relationship bonds as well as teach parents how to set and enforce rules [39, 40].

This study aimed to explore parents’ experiences with the use of the community dialogue approach as a medium to address social norms and attitudes in order to reduce early childbearing. The case study was conducted within a cluster randomised-controlled trial in rural Zambia evaluating community dialogue as part of an intervention package that also included economic support.

## The RISE study

The University of Zambia and University of Bergen, Norway, have implemented the Research Initiative to Support the Empowerment of Girls (RISE) programme in 157 schools and their surroundings in Central and Southern provinces to test whether economic support alone or in combination with community dialogue meetings and youth clubs providing sexuality education, can reduce teenage pregnancy, child marriage and school dropout. In 2016, the trial enrolled approximately 4900 girls who were in grade 7 (average age approximately 14 years). The interventions were provided for 2 years from September, 2016 to November, 2018 [10]. The youth club meetings took place every 2nd week during the school term (i.e. 6 meetings per term), and lasted 60–90 min. Boys in the same grade as the RISE participants were also invited to participate. The topics covered in the youth club included the benefits of education, adolescence and puberty, reproduction myths, communication, early pregnancy and early marriage, gender roles, self-esteem, assertiveness and decision-making, love and infatuation, sexual behaviour and desire, ways to prevent pregnancies and sexually transmitted infections. The most frequent activities were group discussions, plenary discussions, role plays, and debates.

Our study focused on the community and parent meetings which employed a community dialogue approach in promoting supportive community norms around education for girls and postponement of early marriage and early childbearing. Embracing the Freirean approach, RISE used participatory methods to facilitate CC to achieve social transformation. The key results of participatory communication are articulation of awareness raising and commitment to action [26, 27]. The community dialogue meetings included interactive discussions on education, early marriages and the risk of early pregnancies, gender roles and sexual and reproductive health, including myths around modern contraceptives. These community dialogue meetings lasted for approximately 90 min, were held twice per term in the schools in one of the study arms and were open to all interested community members. Most of the meetings started with a film or a role play, and the major part of the meetings was group and plenary discussions where the facilitators were instructed to refrain from teaching but instead ask questions that could make parents and community members reflect on the topic of discussion. Two fictional films were produced and shown in both the youth club and community meetings. The plot in the films took place in a rural Zambian village; one was about the benefits of education and one about the dangers of early pregnancy. Details of the RISE project have been reported elsewhere [10].

## Methods

### Study design

This qualitative study was conducted in November 2018 in six schools in Chibombo district, Central province of Zambia. The schools were selected among schools where the RISE community dialogue was implemented. The selection was convenient based on which schools were holding meetings during the weeks the field work was going on.

#### Data collection methods

We conducted Focus Group Discussions (FGDs) with female and male parents and guardians of RISE trial participants. The FGD participants were purposively selected among parents/guardians who frequently attended the community dialogue meetings and who had children who attended the youth club meetings. Each of the six FGDs had from 6 to 10 participants, and the total sample was forty eight (48).

FGDs provide a context in which participants can feel comfortable to discuss SRH topics and carefully share their experiences and perceptions. Data collection was done by the first author who has postgraduate training in qualitative research. A semistructured interview guide was prepared and piloted. Supervision and support during data collection was provided by the other authors who have experience in conducting qualitative research.

A voice recorder was used to record the discussions. The participants were asked for permission to the use of the voice recorder. All interviews were recorded and later transcribed by the first author. Field notes were also taken.

### Data analysis

Thematic analysis was conducted, and analysis started in the field with familiarization of data through reading and re-reading the notes. Transcripts were critically reviewed by the other authors and codes were agreed on, reflecting meaning units emerging from the data. The coding process was carried out with Nvivo version 11. The first and second authors independently reviewed the codes and categories and then discussed their insights to develop the final themes and sub-themes [41]. The other authors reviewed and approved the final themes.

### Ethical issues

Ethical clearance was obtained from the University of Zambia Biomedical Research Ethics Committee (IRB00001131 of IQRG0000774, reference number 029-06-17). Verbal consent was sought from all study participants before conducting the focus group discussions. Confidentiality was ensured during and after the study by not recording personal details and anonymization of transcripts. The participants in the FGDs were not offered incentives apart from snacks during the discussions.

## Results

### Demographic characteristics of parents

Of all the participants, 85% were female and 15% were males. They were all peasant farmers. From the interviews the following themes emerged (Table 1).

**Table 1:** Major themes and sub-themes

Major themes	Sub-themes
Improved relationship and communication between parents and children	Learning ways to discuss sensitive matters with children
Parents’ support to girls’ education and assertiveness	Awareness of the rights of children
	Allowing children to study
	Awareness of the potential that girl children have
	Parents also going back to school
	Increased sense of collective responsibility
Parents’ perceptions of dialogues on early marriage, pregnancy and school dropout	Good facilitation skills and interactive process
	Some topics being taught too sensitive

## Improved relationship and communication between parents and children

### Learning ways to discuss sensitive matters with children

Most participants said the dialogue enabled them to open up on SRH issues, which were traditionally perceived as secretive. They reported more open discussion on such issues in the community at large and with their children, their spouses and other family members. The dialogue reduced the cultural barriers in communication at all levels. Most parents expressed the view that the dialogues encouraged them to talk more openly about sexual matters and early marriage with their children without fear because the community dialogue meetings were like an ice breaker.

‘Indeed, I used also to fear that if I start explaining these matters of SRH, I would destroy my girl’s mind and she will engage in immoral activities. As we all know that our culture does not allow us to discuss such sensitive matters. Now when they started learning about them and we also started learning the same things, we discussed and I encouraged her to continue with school and not to follow peer pressure’ (FGD 3 male).

The dialogue was seen by most participants as a culturally acceptable way to encourage discussion on sensitive topics between children and parents and other older adults. One participant said:

‘For me, I have seen a lot of change in my child after all the things they learn in youth clubs and the things we learn in community meetings. We learn the same topics in community meetings and the ones the children learn in youth clubs. The children come to explain what they learned and then we discuss with our children and this is a big change for me because long ago I could not discuss issues of SRH with my child’ (FGD 7 female).

Some participants related a sense of discovery about their own ability to change their behaviour towards their children. This can be noted from the comments made by a parent who said:

‘The lessons we learned with our children have opened our eyes. I didn’t know how to start to interact with my child on SRH issues. It was really a far-fetched idea. But when we started community meetings, it gave me the freedom to openly talk with my child. I am now free. There are no secrets now between us.’ (FGD 5 female).

Some parents expressed that open discussions improved their relationship with their daughters:

‘My relationship with my daughter has improved greatly. When she comes from school, we first discuss what she learnt at school and then we discuss what I learnt at community meetings. We both look forward to meet at the end of the day. Unlike long time ago, after school my daughter would first go to play with friends and reach home very late. This time we have a bond, she does not hide anything from me, especially what they learn in youth clubs.’ (FGD 5 female).

The parents acknowledged inadequate knowledge and negative attitudes towards condom and contraceptive use. Nearly all the participants appreciated the correction of misconceptions they had towards condom and contraceptive use in regard to early pregnancy, and how contraceptive methods can contribute to reduce early marriage and school dropout.

## Parents’ support to girls’ education and assertiveness

### Awareness of the rights of children

Participants mentioned the general lack of information on children’s rights as a significant factor in early pregnancy, marriage and school dropout. Most parents had not been aware of children’s rights.

‘I did not know that children have rights because according to our tradition, a child should not question whatever the parents say because that is the culture we have grown up with. For example, when a girl child reaches of age, they are ready for marriage, according to our tradition especially for us women, even the girls themselves know about it and could not resist. But with the coming of the teachings of the RISE on children’s rights, we as parents and the girls have been empowered with knowledge that a girl child’s fate is not marriage but education.’ (FGD 1 female).

### Allowing children to study

One of the ripple effects of knowing the rights of children was that the parents were able to provide an opportunity to children to allocate more time towards school work. Parents reported taking up some of the tasks that children previously had to allow them to do school work.

‘I also did not know that children had rights and responsibilities. When I learnt about the rights of children, we discussed with my child and I gave her time and space to study and do her homework. I have learnt that a child is not supposed to be doing house chores all the time, especially at the expense of schoolwork.’ (FGD 5 female).

Parents further reported that this awareness made them feel that they had become more supportive to their children in general.

‘With the coming of RISE community meetings, it has brought some understanding on children’s rights. We don’t overload our children with home chores anymore. I think we have become better parents now. (Laughing). I think all of you parents can bear witness with me on how we used to lazy around and overload our children with chores. For me it has been my turning point. With all this, I have learnt that I am part of my child’s education.’ (FGD 3 female).

### Awareness of the potential that girl children have

Most study participants agreed that dialogues and watching and discussing the films empowered the parents to help bring about desired changes in the adolescent girls.

‘When I watched the film about Mutinta and Michelo [about the benefits of education], that is when I knew that there is great potential for a girl child to make it in a corporate world. Our tradition, however, always taught us that the pride of a woman is marriage. Now, after watching the film, I learnt that the girl child can complete education and still be of great contribution to the family and the community at large. I will make sure that my child finishes school so that she can take care of me when I grow very old. The idea of marrying my daughter off is a nightmare now.’ (FGD 4 male).

During the interviews with parents, it was reported that marrying the girls off was culturally accepted if she was out of school. They previously believed that once an adolescent girl reaches maturity and has a child, she is supposed to marry. These ideas were challenged in the community dialogue meetings and they claimed that they now perceived that education added to the value of a girl. This would not only be reflected in the higher bride wealth payments they could expect to receive for her, but it would also make her a better wife and mother because of the higher capabilities it conferred on her. The following statement depicts the perceptions of one of the parents:

‘Among the things I learned from RISE, I have seen change in our community. Long ago, it was fine to take young girls into marriages because our culture fully supported the practice. We thought education was just meant for boys only, but now we know that even girls can bring change and bring positive impact in our community as a whole if they get educated. Girl children, if they are educated, do take care of the family much more than the boys. Yes, long ago girls were meant to wash plates and were being taken into marriages very early in exchange for cattle.’ (FGD 1 female)

In a FGD one father said:

‘According to the lessons we learned, I have come to understand that women take care of the whole family if allowed to be educated. Early marriage is not a good thing because a child is not mature enough and she may fail to deliver properly at the hospital due to underage. I have also observed that fistula may occur to underage girls who become pregnant. Working with RISE on the teachings of discouraging early marriage has empowered our children to make better choices in life.’ (FGD 7 male)

The topics discussed in both the community dialogue and youth club meetings were meant to help the parents and the girls themselves discover and build on the potential that girl children have. One of the parents talked about what happened to her daughter:

‘For example, my daughter was proposed marriage by an old man and the man even enticed her by giving her money, but my daughter refused and tore the money and came to report to me. My daughter persuaded me to talk to the man. I met the man when I was with my daughter. She boldly pointed at him as the culprit of the marriage proposal without hesitating. I confronted the man and told him off. He tried to refuse, but I boldly embarrassed him and eventually the man apologised. In those days before the knowledge of children’s rights, this would have been an answered prayer for a fortune. Learning about children’s rights has enlightened me and brought empowerment for our girls and the community at large.’ (FGD 1 female)

### Increased sense of collective responsibility

Most participants agreed that the dialogues were able to create safe and respectful places for discussion and learning. Participants also stated that community dialogue contributed to a sense of community cohesion in addressing early pregnancy, marriage and school dropout. Community problems and priorities were identified and solutions were in turn proposed. One parent said:

‘For me, I have seen that these community meeting teachings have encouraged our children to continue to go to school and has given me power to even discipline any other child in our area who is not behaving well. I am saying so because we now work as one in the community with one motto of girl child education. We watch over each other’s children so that this community could become a better one in terms of girl child education. Unlike a long time ago when my neighbour’s child was in the wrong, I would not be comfortable to talk to the child. For example, if I find any girl in our community aimlessly walking with boys, I intervene. So, it is the same with any other parent. RISE has helped us to take responsibility of our own community. We are now a team with responsibility’. (FGD 6 male).

To address a general lack of information about early pregnancy, marriage and school dropouts, the communities proposed continued sensitisation at household level even when the RISE project community dialogue meetings would wind up.

‘What is so overwhelming is the fact that we learn the same things with my daughter at the community meetings and the youth club, respectively. We actually compete in narrating what was taught and then discuss the way forward. We do all this even with the other children who don’t attend the youth clubs and of course with their father who does not attend the community meetings.’ (FGD 3 male).

### Parents also going back to school

Some participants felt the dialogue had empowered them to take a stand in fighting early pregnancy marriage and school dropout both within their families and throughout the community at large. This made the parents feel they made wiser decisions for their children’s future and their own too. One parent even started going to school herself.

‘My contribution is in a form of testimony, when I watched the film of Mutinta and Michelo that is when I learnt that the sky is the limit. I have decided to go back to school myself and I will be writing my grade nine exams this year together with the adolescent girls.’ (FGD 2 female)

## Parents’ perceptions of dialogues on early marriage, pregnancy and school dropout

### Good facilitation skills and interactive process

The community dialogue meetings frequently generated an interactive process with storytelling amongst participants, and some people recounting emotional, sometimes touching, and sometimes inspiring, personal stories of coping with girls’ behavior in their families. Community members came up with many concrete action plans and reported some success in enacting these plans.

‘The manner in which everything was being taught is very educative, in that it is interactive, involving everyone without intimidation. Furthermore, they taught in our own local language. These facilitators are competent to deliver what they were teaching in the community meetings. It is an eye opener to us because everything that we were taught is also being taught to our children in the girl’s youth club’ (FGD 1 female).

### Some topics being taught were too sensitive to talk about with children

Some participants felt that certain topics their children were taken through in the youth clubs were age-inappropriate and culturally unacceptable, like sexuality, condom and contraceptive use.

‘All the topics are good and educative except the one on condom use which is [too] sensitive. However, given a chance to talk to my daughter, I would tell her to choose what is good for her life because on the condom, it is her life, it is her choice. Again, these condoms are not even safe because they burst and other men have the habit of tearing them deliberately’ (FGD 4 female).

Parents were of the view that age should be taken into consideration to get desired results from the SRH discussions. They testified that some children were hearing about condoms and contraceptive for the first time.

‘In my view, I think it is not a good idea to talk about condoms and contraceptives to our young children. For me, when I learnt about it, I didn’t talk about it with my daughter because it would have been like encouraging her to sleep with boys. If I tell her about condom use, it would lead to practice’ (FGD 2 male).

## Discussions

We found, in line with previous studies from sub-Saharan Africa [3, 32, 38, 42, 43], that communication in SRH matters between parents and adolescents is a taboo, leading to such communication occurring infrequently and being compounded by discomforts. The community dialogue meetings, particularly the films and the following discussions, enabled many of the parents to communicate with their adolescents about SRH needs, early pregnancy and marriage, and to reflect on how they could support their children to do well in school. However, it is worth mentioning that a minority of the participants expressed negative views regarding communication with adolescent children about sexuality and contraceptive use.

The parents’/guardians’ statements indicated that the main reasons for the limited communication about SRH were conservative norms around sexuality, limited parent reproductive knowledge, embarrassment and fear that such communication would encourage sexual activities, and this is in line with findings from several countries in sub Saharan Africa [32, 38, 40, 42, 44]. Paulo Freire postulated that dialogue provides opportunities for critical thinking, questioning of assumptions, and development of new ideas among group participants [28, 29]. Our findings suggest that the facilitators of the community dialogue managed to initiate such processes and helped communities take up new practices to better support their adolescents in making SRH related decisions and other decisions about their life. Future studies should ideally be conducted to explore the longterm impact of community dialogue meetings.

Earlier research suggests that communication between parent and child should evolve over time because the social abilities of the child develop and change during the adolescent years [3, 32, 38]. Zambia has made efforts to offer a comprehensive sexuality education curriculum in schools. However, efforts to achieve parental involvement in adolescent SRH information have been more limited [43]. The findings of this study indicate that parental involvement and direction on SRH matters is feasible.

It is worth noting that parents talked about the community dialogue meetings as trainings although they were meant to constitute an interactive communication process of sharing information between equal parties, in order to reach a common understanding and consensus to address a specific issue. Although the repeated use of the words “teaching” and “training” could possibly indicate that the participants perceived the project to promote a specific message, it is more likely to reflect that they were used to referring to meetings taking place at school as “teaching” since monitoring of the meetings indicated that most of the facilitators successfully managed to avoid instructing parents. Furthermore, we cannot rule out that the participants sounded more enthusiastic about the community dialogue meetings than they actually were because they may have perceived it to be socially desirable during the FGD, particularly since it was done at the school where the community meetings took place. Considering that the moderator (the first author of this paper) was unknown in the study communities and comes from another province, this may have made some participants less confident about sharing their opinion, However, since she was not part of the team that had implemented or monitored any of the RISE interventions and she encouraged everyone to be free, we do not expect this risk to be high. We believe that trustworthiness of the findings was enhanced by two authors coding the data independently and agreeing on the themes.

## Conclusion

The community dialogue meetings appeared to contribute to parents becoming aware of the need to make sure adolescents are well informed about SRH to make good decisions and thus becoming willing to communicate with their adolescent children about SRH topics. They expressed appreciation over the meetings and indicated that they resulted in an increased feeling of collective responsibility for children’s welfare. However, topics like contraceptive and condom use were found to be challenging, and some—after attending several community meetings—still thought they were inappropriate to discuss with adolescents.

## Data Availability

The interviews analyzed during the current study are available from the corresponding author on reasonable request.

## Abbreviations

AIDS: Acquired Immunodeficiency Syndrome
CC: Community conversation
FGD: Focus group discussion
HIV: Human Immunodeficiency Virus
RISE: Research Initiative to Support the Empowerment of Girls
SRH: Sexual and reproductive health
